# Arrhythmogenic Mechanisms in Hypokalaemia: Insights From Pre-clinical Models

**DOI:** 10.3389/fcvm.2021.620539

**Published:** 2021-02-03

**Authors:** Gary Tse, Ka Hou Christien Li, Chloe Kwong Yee Cheung, Konstantinos P. Letsas, Aishwarya Bhardwaj, Abhishek C. Sawant, Tong Liu, Gan-Xin Yan, Henggui Zhang, Kamalan Jeevaratnam, Nazish Sayed, Shuk Han Cheng, Wing Tak Wong

**Affiliations:** ^1^Tianjin Key Laboratory of Ionic-Molecular Function of Cardiovascular Disease, Department of Cardiology, Tianjin Institute of Cardiology, Second Hospital of Tianjin Medical University, Tianjin, China; ^2^Faculty of Health and Medical Sciences, University of Surrey, Guildford, United Kingdom; ^3^Faculty of Medicine, Newcastle University, Newcastle upon Tyne, United Kingdom; ^4^Li Ka Shing Faculty of Medicine, The University of Hong Kong, Hong Kong, China; ^5^Second Department of Cardiology, Laboratory of Cardiac Electrophysiology, Evangelismos General Hospital of Athens, Athens, Greece; ^6^Division of Cardiology, Department of Internal Medicine, State University of New York at Buffalo, Buffalo, NY, United States; ^7^Lankenau Institute for Medical Research and Lankenau Medical Center, Wynnewood, PA, United States; ^8^School of Physics and Astronomy, The University of Manchester, Manchester, United Kingdom; ^9^Stanford Cardiovascular Institute, Stanford University School of Medicine, Stanford, CA, United States; ^10^Institute for Stem Cell Biology and Regenerative Medicine, Stanford University School of Medicine, Stanford, CA, United States; ^11^Department of Medicine, Division of Cardiology, Stanford University School of Medicine, Stanford, CA, United States; ^12^Department of Biomedical Sciences, College of Veterinary Medicine and Life Science, City University of Hong Kong, Hong Kong, China; ^13^State Key Laboratory of Marine Pollution (SKLMP), City University of Hong Kong, Hong Kong, China; ^14^Department of Materials Science and Engineering, College of Science and Engineering, City University of Hong Kong, Hong Kong, China; ^15^School of Life Sciences, Chinese University of Hong Kong, Hong Kong, China

**Keywords:** hypokalaemia, potassium, cardiac arrhythmia, conduction, repolarization

## Abstract

Potassium is the predominant intracellular cation, with its extracellular concentrations maintained between 3. 5 and 5 mM. Among the different potassium disorders, hypokalaemia is a common clinical condition that increases the risk of life-threatening ventricular arrhythmias. This review aims to consolidate pre-clinical findings on the electrophysiological mechanisms underlying hypokalaemia-induced arrhythmogenicity. Both triggers and substrates are required for the induction and maintenance of ventricular arrhythmias. Triggered activity can arise from either early afterdepolarizations (EADs) or delayed afterdepolarizations (DADs). Action potential duration (APD) prolongation can predispose to EADs, whereas intracellular Ca^2+^ overload can cause both EADs and DADs. Substrates on the other hand can either be static or dynamic. Static substrates include action potential triangulation, non-uniform APD prolongation, abnormal transmural repolarization gradients, reduced conduction velocity (CV), shortened effective refractory period (ERP), reduced excitation wavelength (CV × ERP) and increased critical intervals for re-excitation (APD–ERP). In contrast, dynamic substrates comprise increased amplitude of APD alternans, steeper APD restitution gradients, transient reversal of transmural repolarization gradients and impaired depolarization-repolarization coupling. The following review article will summarize the molecular mechanisms that generate these electrophysiological abnormalities and subsequent arrhythmogenesis.

## Introduction

Hypokalaemia is the most common electrolyte abnormality found in hospitalized patients (1) and therefore represents an important cause of arrhythmias and associated mortality observed in clinical practice (2). It is commonly observed in patients with pre-existing heart conditions (3–5). Hypokalaemia manifests, in order of decreasing likelihood, due to (i) increased K^+^ loss, (ii) transcellular K^+^ shift into cells or (iii) reduced dietary K^+^ intake. Increased loss of K^+^ mostly occurs secondary to the use of diuretics or laxatives, or from diarrhea. Transcellular shift of K^+^ into cells can be caused by medications, such as β_2_ receptor agonists (6), hormonal abnormalities, or metabolic alkalosis (7). Decreased intake can develop in conditions such as anorexia, dementia or reduced appetite from malignancy.

The following features are observed on the electrocardiogram (ECG) during hypokalaemia: ventricular premature complexes (VPCs), prolonged QT interval, ST segment depression and the appearance of a U wave (8). Extracellular potassium concentration ([K^+^]_o_) is negatively correlated with the development VPCs, with each unit decrease in [K^+^]_o_ (mM) corresponding to a 28% increased risk of VPCs (9, 10). A potentially life-threatening form of ventricular tachycardia (VT) termed *torsade de pointes (TdP)* also manifests in hypokalemia (11), which in turn can degenerate into ventricular fibrillation (VF) and sudden cardiac death (12). Other cardiac rhythm abnormalities induced by hypokalaemia include atrial fibrillation (13) and atrial flutter (14).

**Figure 1:**
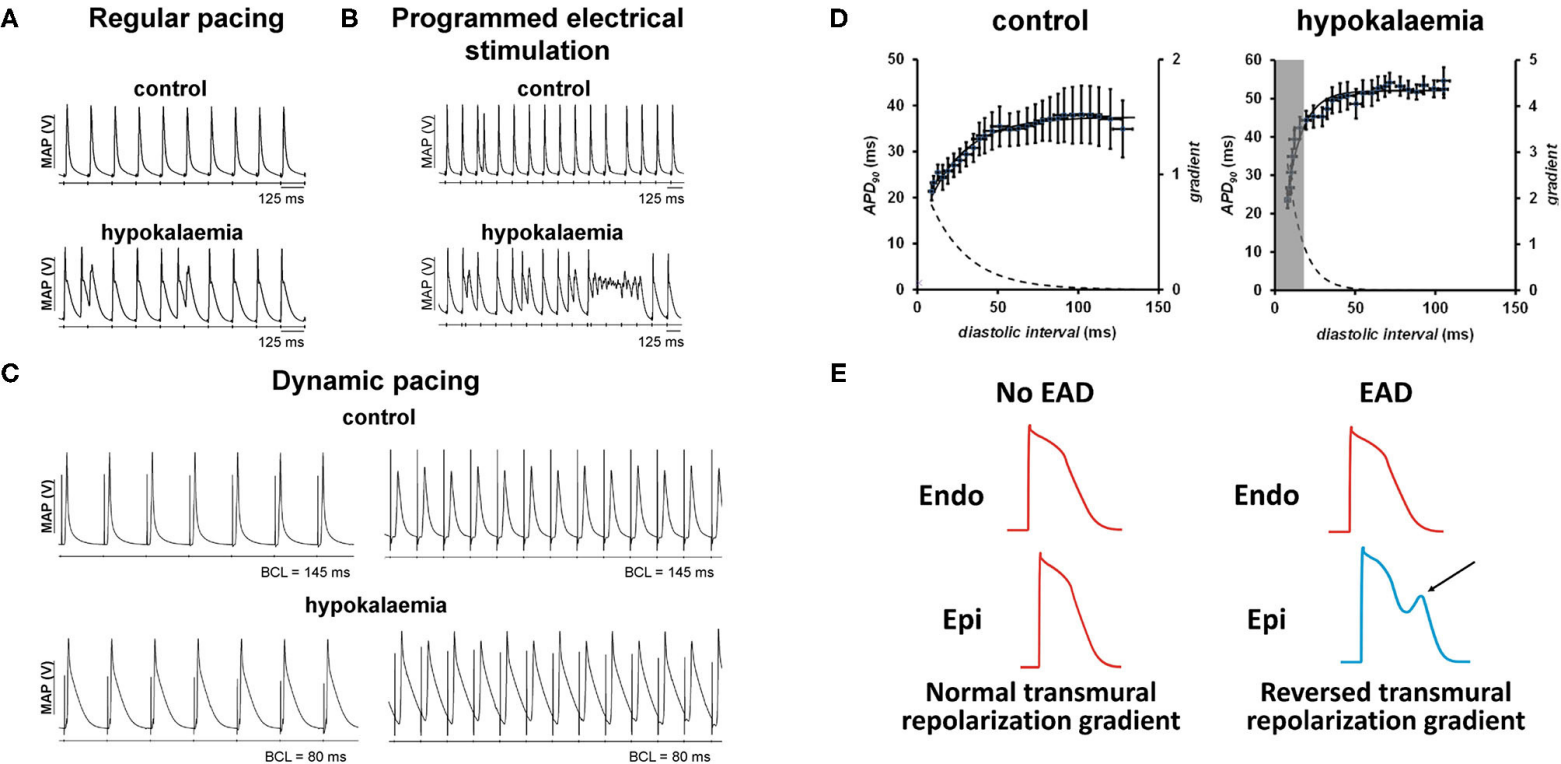
**(A)** Hypokalaemia prolongs APDs, which predisposes to triggered activity (left). This AP prolongation and reduced refractoriness together form a re-entrant substrate. **(B)** The use of programmed electrical stimulation can reliably provoke ventricular arrhythmias (right). [Traces reproduced from (25) with permission]. **(C)** Hypokalaemia exacerbates APD alternans at fast heart rates during dynamic pacing [Traces reproduced from (24) with permission]. **(D)** The onset of alternans can be explained by steep APD restitution. **(E)** Development of early afterdepolarizations in one region (arrow) can exacerbate transmural differences in repolarization time, predisposing to unidirectional conduction block and reentry [trace partly adapted from (26) with permission].

Animal models, particularly guinea pigs (15–21) and mice (22–24), have provided much insight into the detailed mechanisms underlying hypokalaemia-induced arrhythmogenicity. In these models, arrhythmic activity has been observed during regular pacing (Figure 1A), programmed electrical stimulation that delivers S1S2 pacing (increasing premature S2 stimuli delivered following trains of regular S1 stimuli) (Figure 1B) and dynamic pacing (trains of regular S1 stimuli of decreasing basic cycle length) (Figure 1C). The review article aims to consolidate pre-clinical findings on the electrophysiological mechanisms underlying hypokalaemia-induced arrhythmogenicity.

## Basic Electrophysiology: Pre-Clinical Lessons From Small Animal Models (Mice, Rabbit, and Guinea Pigs)

Whether serving as a disease model for pharmaceutical purposes or toxicology, the use of animal models as fundamental building blocks has enabled rapid advances in biomedical knowledge (27). This is no different in cardiology, with mice, rabbit and guinea pigs considered to be the most frequently used animal models in experimental cardiac electrophysiology (28). However, despite similarities in cardiac ion channel distribution, salient differences in electrophysiological results are still observed between small animal species, especially within the context of hypokalaemia.

### Triggered Activity Can Arise From Afterdepolarizations

At the cellular level, reduction in [K^+^]_o_ is expected to shorten the time course of repolarization by increasing the K^+^ electrochemical gradient across the cell membrane. Recent experiments conducted in rabbit hearts showed that hypokalaemia activated the apamin-sensitive small-conductance calcium-activated potassium current (I_KAS_) to shorten action potential durations (APD), thereby preserving repolarization reserve (29). However, prolonged APDs are observed because of I_Kr_, I_K1_, I_Ks_, and I_to_ inhibition (30–34). These repolarization abnormalities explain the electrocardiographic QT interval prolongation observed in clinical practice (35). In a similar hypokalaemic *in vivo* rabbit model, prolonged exposure to reduced [K^+^]_o_ was also found to be significantly correlated with decreased HERG channel density due to its internalization and subsequent degradation, which may play a major role in APD prolongation (36). Recently, reduced Na^+^/K^+^-ATPase currents have been identified as a contributory mechanism toward prolonged repolarization (37, 38). Normally, Na^+^ and Ca^2+^ handling is closely coupled via the sodium-calcium exchanger (NCX), which uses the electrochemical gradients of both ions to exchange three Na^+^ for 1 ${\text{Ca}}_{.}^{2+}$

A change in the morphology of the action potential, such as in triangulation reflected by an increase in the APD_90_-APD_50_ difference, is thought to increase the likelihood of inward current re-activation that in turn produces triggered activity over the terminal phases of action potential repolarization (16, 39). More severe reductions in [K^+^]_o_ can induce Ca^2+^ overload due to a combination of suppressed Na^+^-K^+^-ATPase activity, reversal of transport by the NCX, and reduced intracellular ATP concentrations (40, 41).

Afterdepolarizations refer to the oscillations in the membrane potential before the next action potential. They can occur early (early afterdepolarizations, EADs) or late (DADs, delayed afterdepolarizations). EADs can be subdivided into those that occur during phase 2 and phase 3. Hypokalaemia can generate both EAD types by distinct mechanisms. APD prolongation increases the susceptibility to phase 2 EADs because of a wider window over which the L-type Ca^2+^ channels can be re-activated (42, 43). Ca^2+^ overload can promote EADs during phase 3 of the action potential (and during phase 2 in some species), thereby activating the NCX to mediate Na^+^ entry (44, 45). Recent experiments in rabbit hearts showed that when combined with increased beta-adrenergic drive, I_KATP_ can be activated, leading to heterogeneous APD shortening and the subsequent generation of late phase 3 EADs in the presence of enhanced Ca^2+^. Intracellular Ca^2+^ accumulation can promote DADs. Isolated, perfused ventricular muscle in guinea pig (46) and rabbit (47) hearts have exhibited DADs in severe, experimental hypokalaemia.

Both EADs and DADs can lead to triggered activity (Figure 1A), thereby initiating arrhythmic activity and producing a sustained tachycardia upon encountering favorable reentrant substrates (48, 49). Such substrates can be revealed by programmed electrical stimulation (PES) (Figure 1B) or dynamic pacing (Figure 1C). Dynamic pacing can unmask APD alternans at short basic cycle lengths (BCLs), which can be explained by steep restitution in hypokalaemia compared to control conditions (Figure 1D). EADs, DADs or triggered activity can themselves increase the spatial heterogeneity in repolarization as well as areas of slowed conduction. In other words, triggers of arrhythmias may themselves create the substrates for re-entry (50), as demonstrated recently in modeling studies (51). Normally, endocardial APD is longer than epicardial APD resulting in a normal repolarization gradient (Figure 1E, left). When an EAD (arrow) develops, epicardial APD will be longer than endocardial APD, causing a reversal in the transmural repolarization gradient (Figure 1E, *right*) that is potentially arrhythmogenic (16, 17, 20).

### Reentry Is Due to Static and Dynamic Abnormalities in Repolarization, Refractoriness and Conduction

Numerous static and dynamic re-entrant substrates contribute to increased arrhythmogenicity in hypokalaemia (52).

#### Repolarization: Steep Spatial Gradients

The most important experimental finding consistently observed across the different species during hypokalaemia is non-uniform prolongation of repolarization, be it when comparing the left (LV) and right ventricle (RV), epicardium and endocardium, or apex and cardiac base (16, 53). Spatial differences in repolarization are thought to increase the risk of unidirectional conduction block, a prerequisite for circus-type or spiral wave reentry (54). Such spatial variations in repolarization may be present during regular pacing and further exacerbated following triggered activity, thereby enhancing arrhythmic risk. In guinea pig hearts, greater APD_90_ prolongations were seen in the RV epicardium relative to the LV epicardium (16, 53). These APD differences were attributed to differing expression patterns and levels of ion channels, in particular higher density of I_K1_ channels in the LV compared to in the RV (55, 56). The consequence of RV APD_90_ prolongation during hypokalaemia is an increased RV-LV transepicardial APD_90_ difference compared to control during both regular and S1S2 pacing (16), which partly underlies the capacity of VPCs to induce sustained VT (57, 58).

In addition to transepicardial repolarization gradients, transmural gradients may contribute to arrhythmogenesis in hypokalaemia. Experimental data obtained from mouse hearts have been conflicting, as pointed out previously (16). LV epicardial and LV endocardial APD_90_ difference was found to be either unaltered (59) or reduced (23, 43). In guinea pig hearts, there was no demonstratable APD_90_ difference between the epicardium endocardium under either normokalaemic or hypokalaemic conditions. Moreover, transient alterations in transmural repolarization gradients have been explored in mouse hearts (60). It was shown that the S2 stimulus proportionally decreased epicardial and endocardial APD_90_. After the following S3 stimulus, endocardial APD_90_ decreased more sharply than did epicardial APD_90_, albeit the former recovered after S4 stimulation.

#### Repolarization: Steep APD Restitution Gradients and APD Alternans

**Figure 2:**
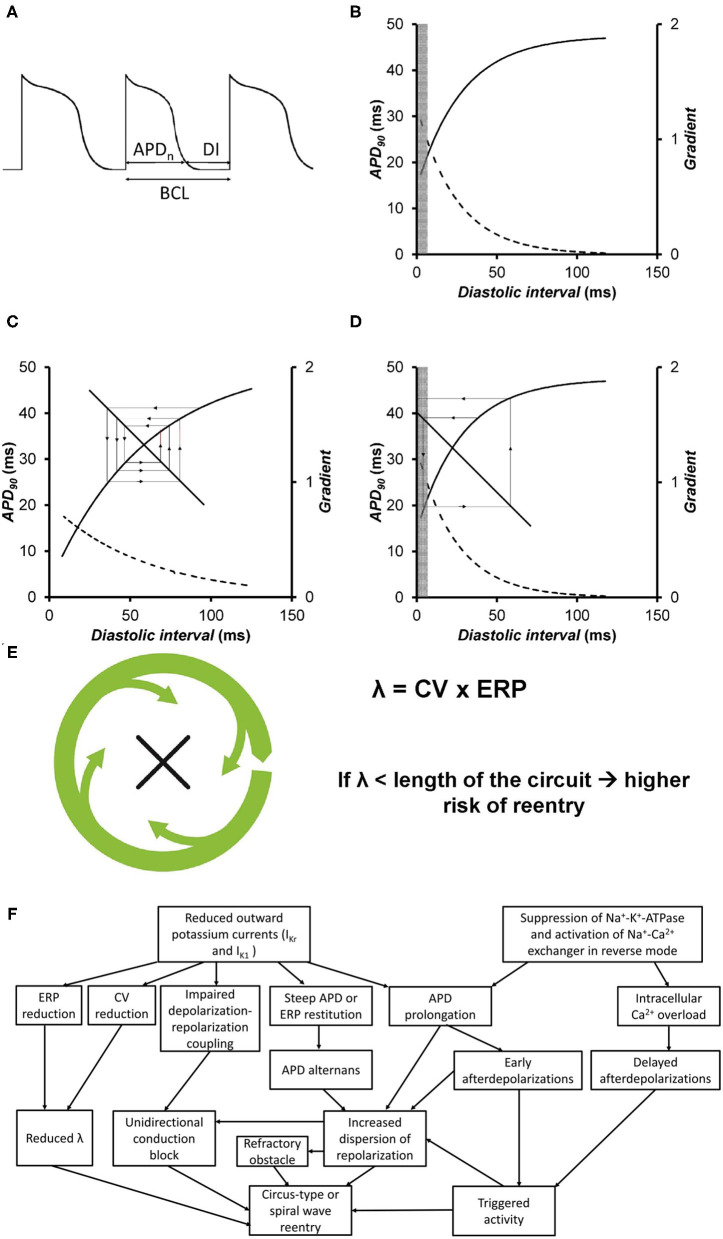
**(A)** Voltage trace showing the relationships between action potential duration (APD), diastolic interval (DI), and basic cycle length (BCL). **(B)** An APD restitution curve describes the relationship between the APD and the previous diastolic interval (solid line). The gradients of the curve are represented by the broken line. The values of DIs at which such gradients are >1 are represented by the gray box. **(C)** APD restitution curve plotting APD against the previous DI (solid line) along with their gradients (broken line). The values of DIs with gradients >1 are represented by the gray box. The cobweb plot shows that when the APD restitution gradient is <1, a stable equilibrium point is produced on successive beats. **(D)** APD restitution curve plotting APD against the previous DI (solid line) along with their gradients (broken line). The values of DIs with gradients >1 are represented by the gray box. The cobweb plot shows that when the APD restitution gradient is >1, an unstable equilibrium point is produced on successive beats, eventually leading to conduction block. Reproduced from (52) with permission. **(E)** Circus-type reentry depends on the wavelength of excitation, given by the product of conduction velocity and effective refractory period [Figures adapted from (26) with permission]. **(F)** Summary of different electrophysiological mechanisms that are responsible for triggered activity and reentry in hypokalaemia.

The relationships between APD, the diastolic interval (DI) and basic cycle length are detailed in Figure 2A. The relationship BCL = APD + DI can be shown graphically as a straight line with a gradient of −1. The original descriptions of alternans were based on a graphical method that related them to restitution of APD (61). APD restitution is the APD abbreviation that occurs when heart rate is increased and reflects an adaptive response to maintain a period of diastole, allowing blood to refill in the cardiac chambers. In a normal APD restitution curve (Figure 2B), APD is plotted against the previous DI. This relationship can be represented by the equation APD_n+1_ = f (DI_*n*_), where f is the function relating the new APD to its previous DI. When DI shortens, APD also shortens to accommodate. The region for long DIs is almost flat, whereas the region at short DIs is steep.

The restitution gradient reflects the recovery of the different ion channels that are activated during action potential generation. Na^+^ channels show the fastest inactivation kinetics and recover quickly, and their effects on restitution are observed mostly at the shortest DIs. The Ca^2+^ channels recover at a slower rate compared to Na^+^ channels, and their effects are observed at longer DIs. Because these channels mediate much of the transmembrane currents during the action potential plateau, they affect APD restitution greatly. K^+^ channels have the slowest recovery rates compared to Na^+^ and Ca^2+^ channels and their effects are therefore mostly observed at long DIs. An important property of K^+^ channels is their reverse use dependence, in which increasing use leads to a lower level of channel blockade (62). As hypokalaemia inhibits K^+^ channels, its effects are most prominent at long DIs, which may occur during a compensatory pause following an ectopic beat, and bradycardia. In hypokalaemia, due to the APD prolongation, the DIs can engage the steeper portion of the restitution curve even when heart rate is normal.

Cobweb plots can be used to illustrate the stability of beat-to-beat alternations in APD (Figures 2C,D). In the original formulation, it is assumed that the DI depends on the preceding APD. The line of the equation, DI = BCL–APD, represents the feedback mechanism, where DI is inversely related to APD. If APD is longer, then the next DI is shorter. The APD equilibrium point at each BCL is located at the intersection between this line and the restitution curve. A sudden increase in heart rate, as reflected by a decrease in BCL, leads to shortening of APD. Under normal conditions, the restitution gradient is <1. With a perturbation leading to a small decrease in DI, the next APD decreases. For the next beat, the DI increases, but to a value smaller than the original DI. Each iteration leads to a smaller beat-to-beat difference in APD and DI, until eventually a stable point is reached (Figure 2C). In hypokalemia, the restitution gradient is steeper at the same range of DIs (63). Each iteration leads to a successive increase in the beat-to-beat variation in APD, leading to 2:1 block (Figure 2D). A special case occurs if the restitution gradient is exactly 1, in this case, alternans do not converge or diverge, and become stable.

The appearance of APD alternans has been associated with steeper APD restitution. However, it should be stressed that restitution is not the only factor that determines the presence or absence of alternans. Thus, other factors such as electronic and memory effects can suppress APD alternans even when the APD restitution gradient is >1 (64). Moreover, normally APD is closely coupled to the effective refractory period (ERP). Yet, APD is prolonged but ERP is shortened in hypokalaemia. Thus, APD restitution may not accurately predict the onset of alternans in this situation and VERP restitution may be a better indicator (18). Conversely, APD alternans can occur when the APD restitution gradient is <1 when restitution-dependent mechanisms are present (65). However, these effects have not been studied in detail for hypokalaemia. Finally, the relationships between repolarization dynamics, membrane excitability and cardiac memory are complex and warrants further study (66–71).

Electrical restitution can generally be assessed by two stimulation protocols: dynamic pacing and S1S2 pacing measure the steady-state response and the intermediate response, respectively, of the myocardium to a change in the basic cycle length (BCL). S1S2 pacing has the advantage of safety because pacing at a high heart rate is not required (19, 72, 73), albeit this method cannot assess beat-to-beat variations, that is, alternans, in action potential properties. In contrast, dynamic pacing can induce myocardial ischaemia (74, 75), but can be used experimentally to quantify alternans. In mice, greater amplitudes of epicardial APD_90_ alternans associated with increased maximum APD_90_ restitution gradients were observed during dynamic pacing in hypokalaemia compared to in normokalaemia (Figure 1D) (63). Endocardial APD_90_, maximum APD_90_ restitution gradients and DI_crit_ were not altered (23, 63). However, guinea pig hearts showed significant differences, such as increased endocardial APD_90_ restitution gradients (18) and APD_90_ alternans despite shallower APD_90_ restitution gradients (18). Recent experiments in mouse hearts have further separated the roles of abnormal electrical restitution from other electrophysiological substrates in hypokalaemia (24). Moreover, these data provide the proof-of-concept that restitution can be assessed by both dynamic and S1S2 pacing procedures with largely agreeable restitution parameters.

#### Reduced Refractoriness and Steep ERP Restitution

The refractoriness of the myocardium, which can be measured experimentally as the effective refractory period (ERP), is an important determinant of the likelihood of reentry for the following reasons. Firstly, a decrease in the excitation wavelength, λ [conduction velocity (CV) × ERP] increases the number of reentry circuits available within the myocardium (Figure 2E) (76). Secondly, an increase in the critical interval given by APD–ERP would prolong the time window during which re-excitation can take place, potentially by reactivation of inward Na^+^ and Ca^2+^ currents (59). Furthermore, reduced ERP can decrease the core size around which a spiral wave can meander (77). Shortening of ERP is observed during hypokalaemia despite concomitant APD prolongation. Studies in mouse and guinea pig hearts showed that LV epicardial and endocardial ERPs were decreased by similar extents (16, 23). Though debatable, this ERP shortening was found to be associated with excessive hyperpolarization of the resting membrane potential in ventricular cardiomyocytes. This subsequently results in increased activation of fast Na^+^ channels, leading to a more pronounced action potential amplitude and an increased upstroke velocity during the depolarization phase (17). Under normokalaemic conditions, the critical opening for LV re-excitation is narrow, rendering the induction of re-excitation highly unlikely. Therefore, it is no surprise that prolongation of the critical interval from reduced [K^+^]_o_ is associated with an increased likelihood of sustained triggered activity over terminal repolarization (16). Recent experiments in guinea pigs demonstrate a contributory role of steep ERP restitution in predisposing the tissues to the generation of alternans and reentry (18).

#### Conduction Slowing

Conduction velocity (CV) is governed by Na^+^ channels and gap junctions (78). Hypokalaemia is known to decrease CV in the atria, atrioventricular node, Purkinje fibers and the ventricles (16, 79). The underlying mechanism is thought to involve depressed membrane excitability from membrane depolarization, increased threshold potential for Na^+^ channel activation and increased membrane resistance (80, 81). Enhanced stimulation threshold, decreased LV to RV transepicardial and LV epicardial to endocardial transmural CVs were all observed in guinea pigs during both regular and S1S2 pacing (16). In contrast, local epicardial and endocardial CV as well as transmural CV were not altered in hypokalaemic mouse hearts (59).

### Impaired Activation-Repolarization Coupling and Other Arrhythmogenic Factors

Activation-repolarization coupling is an intrinsic property of the myocardium, allowing local APD values to be adjusted to conduction slowing at different myocardial sites along the path of the propagating action potential (21). This effect has been attributed to modulation of APD in neighboring cardiomyocytes by gap junction conduction, which would reduce regional differences in APD (82). Normally, the APD difference between the RV and LV is minimized by delayed LV activation, an effect that is impaired by hypokalaemia (21).

It is worth noting that arrhythmogenicity is stimulation site-dependent. Experiments in guinea pig hearts showed that ventricular arrhythmias were readily inducible upon LV stimulation, whereas RV stimulation failed to induce arrhythmic events (15). This observation can be attributed to interventricular differences in ion channel expression. Thus, larger *I*_K1_ is found in the LV compared to in the RV, which would be expected to shorten APDs and therefore ERPs to greater extents in the LV. A steep repolarization gradient between the epicardium and endocardium, and between the LV and RV, can lead to a block of an action potential, favoring reentry. All of the above electrophysiological mechanisms underlying arrhythmogenesis in hypokalaemia are summarized in Figure 2F.

## Basic Electrophysiology: Larger Animal Models—Canine, Cat and Sheep

It is important to note the fundamental relationship and differences between body weight and various cardiovascular parameters across all types of laboratory animals. An equation encapsulating this concept was coined in 1979 as heart weight (HW (g) = 6.0 × BM^0.98^) and P-R interval (PR (ms) = 53 × BM^0.24^) where BM is body mass in kg (83). Such differences are reinforced in electrophysiology, where small rodents are found with significantly shorter APD than humans due to lack of a prominent plateau phase found in cardiomyocytes (84–86). Therefore, the rabbit myocardium presents a more representative model of the human heart. Despite this similarity, important inter-species variations remain especially when K^+^ handling is examined. Cardiac K^+^ channel expression is significantly different between rabbits, guinea pigs and humans, accounting for the increased susceptibility to ventricular fibrillation in rabbit hearts, as well as the reduced transient outward current and large slow component of the delayed rectifier current in guinea pigs (87).

Furthermore, it is imperative to consider the potential usage of other relevant cardiovascular animal models. Similar to rabbit models, canine heart models show similar cardiac ion channel distribution with human hearts, making them suitable for the study of ion-channel-related mechanisms (e.g., repolarization and depolarization mechanics) and arrhythmic drug effects. Moreover, canine heart models have a much more comparable APD, sino-atrial node activity, Purkinje fiber distribution and activation sequence to humans (88–90). In contrast, goat and horse models have also shown to be suitable for the study of atrial fibrillation given the ease of obtaining ECG recordings (28, 91, 92). Regardless, mainly canine, cat and sheep models have been used to investigate electrophysiological changes in hypokalaemia.

Canine and sheep models were similar to smaller animal models with regards to an observed reduction in conduction velocity during hypokalaemia across the cardiac conduction system (atria, atrioventricular node, Purkinje fibers and the ventricles) (93, 94). The underlying mechanism was thought to involve depressed membrane excitability from membrane depolarization, increased threshold potential for Na^+^ channel activation and increased membrane resistance (80). However, further experiments have shown differing effects of hypokalaemia on epicardial vs. endocardial APD parameters (95) as well as regional differences in repolarization in canine hearts, due to greater I_Ks_ and I_to_ in RV compared to in the LV (96, 97). This shows that both the interlayer restitution gradient and transepicardial APD difference constitute viable pathways for arrhythmogenesis.

## Differential Effects of Hypokalaemia on Distinct Cell Types

Arrhythmogenic mechanisms in atrial and ventricular cell types can differ. For example, EADs in ventricular cardiomyocytes and tubulated atrial cardiomyocytes are attributed to Ca^2+^ overload (98). However, phase 3 EADs in untubulated atrial cardiomyocytes are instead linked to the reactivation of non-equilibrium Na^+^ current and are driven by membrane hyperpolarization and short action potential configurations (98). Furthermore, hypokalaemia induces Ca^2+^ overload in ventricular cardiomyocytes by reduced pumping rate of the Na^+^-K^+^-ATPase leading to subsequent Na^+^ accumulation (37). Moreover, structurally and functionally different small conductance Ca^2+^-activated K^+^-channel (KCa2) inhibitors, ICA, AP14145, and AP30663, exerted anti-arrhythmic effects in hypokalaemic guinea pig hearts (99). In contrast, KCa2 blockade was found to be pro-arrhythmic in rabbit hearts (29), the reasons for which may be attributed to species differences or variations in the pharmacological agents used (ICA, AP14145, and AP30663 vs. apamin) (99). Both AP14145 and AP30663 can inhibit the late Na^+^ current at higher concentrations (100). Indeed, the increase in intracellular Ca^2+^ can activate Ca^2+^-calmodulin-dependent kinase to increase the activity of the late Na^+^ channel (38). Hypokalaemia can also cause conduction abnormalities in the cardiac conduction system, although not to the same extent as hyperkalaemia. Thus, it can cause slowed conduction of action potentials through the atrioventricular node in canine (94, 101) and rabbit hearts (81), an abnormality that has also been reported in humans (102).

## Bridging Over From Basic to Clinical Electrophysiology

Human cardiac models tend to have differences in repolarization reserve when compared to animal models, depending on cardiac miRNA levels for ion channel subunit production (103). Utilizing human induced pluripotent stem cell-derived engineered heart tissue can overcome this human-to-animal model gap to better simulate physiological outcomes in humans (104). While there is a limited understanding specifically on the implications of steep AP restitution gradients within the context of human hypokalaemia, the heterogeneity of APD restitution slopes have been proposed as a substrate for arrhythmogenesis in a whole-heart modeling study (105). This phenomenon was subsequently confirmed by the introduction of the Regional Restitution Instability Index (R2I2) by Nicholson and colleagues (106, 107).

## Hypokalaemia in the Clinical Context

The importance of understanding the underlying mechanisms during hypokalaemia resides in its relationship with the development cardiac arrhythmias in various clinical conditions. Hypokalaemia is associated with increased risks of atrial fibrillation amongst hospitalized patients (108). Moreover, hypokalaemia is common in patients presenting with VT/VF, and those with severe hypokalaemia have found to be associated with preceding gastrointestinal illness, higher doses of diuretics (109), use of drugs such as anti-depressants (110), as well as post-operative settings (111). In patients with implantable cardioverter-defibrillators (ICDs), hypokalaemia but not hyperkalaemia has been linked with increasing risk of recurrent ventricular tachyarrhythmias and appropriate ICD therapies (112). However, it should be stressed that the relationship between hypokalaemia and adverse outcomes is complex, in that it may or may not be an independent predictor of mortality (113) and that its correction may not lead to better outcomes in hospitalized patients (114). Moreover, altered repolarization correlates with prolonged QTc and T_peak_-T_end_ intervals in pre-clinical experimental studies (99). Both ECG indices have been reported to provide predictive value for arrhythmic risk stratification in the clinical context of acquired long QT syndrome for humans (115). Indeed, in a Chinese cohort of patients with acquired long QT syndrome, random survival forest analysis identified hypokalaemia as the second most important variable after cancer for predicting all-cause mortality (116).

In heart failure, the use of diuretics and activation of the renin-angiotensin system are the predominant causes of hypokalaemia (117). Ventricular arrhythmias, particularly non-sustained VT, are common (118, 119), involving both triggered and re-entrant arrhythmias have been described (120–122). A recent meta-analysis suggested a strong inverse association between serum K^+^ channel concentration and ventricular arrhythmias in patients with myocardial infarction (123). In a large animal model of chronic post-myocardial infarction fibrosis, hypokalaemia revealed vulnerable electrophysiological substrates, which highlighted the importance of conduction slowing over repolarization instability in its arrhythmogenesis (124). Thus, clinical decision-making should take into consideration hypokalaemia as a common side effect of diuretics in patients with prior myocardial infarction (125, 126). In emergency settings, serum K^+^ concentrations on admission alone or together with the co-existing Thrombolysis in Myocardial Infarction (TIMI) risk score was shown to predict more accurately short- and long-term risk of malignant ventricular arrhythmias respectively (127, 128). Moreover, hypokalaemia is not only a risk factor for VT/VF in the acute phase of ST-segment-elevation myocardial infarction (STEMI), but is also associated with VF before primary percutaneous coronary intervention (129). Finally, hypokalaemia exerts pro-arrhythmic effects in congenital long QT syndrome, such as in the context of salt-wasting nephropathy (130). In otherwise silent mutational carriers, it can reveal a long QT phenotype (131, 132). In such patients, K^+^ supplement can protect congenital LQTS patients or silent carriers against the development of VT/VF (133, 134).

## Conclusion

This article reviewed the electrophysiological mechanisms of triggered and re-entrant arrhythmogenesis in hypokalaemia, in which the data were largely derived from pre-clinical animal models. Prolonged repolarization can cause EADs, and Ca^2+^ handling can lead to the development of both EADS and DADs, leading to triggered activity. Reduced conduction velocity, prolonged repolarization, increased dispersion of repolarization, reduced refractoriness, steep APD restitution gradients, transient reversal of transmural repolarization gradients and impaired depolarization-repolarization coupling, all collectively contribute to reentrant arrhythmogenesis.

## Author Contributions

GT and WW: drafting of manuscript, revision of manuscript, preparation of figures, and data interpretation. All other authors: drafting of manuscript, revision of manuscript, and data interpretation.

## Conflict of Interest

The authors declare that the research was conducted in the absence of any commercial or financial relationships that could be construed as a potential conflict of interest.
